# Liraglutide Protects Against Brain Amyloid-β_1–42_ Accumulation in Female Mice with Early Alzheimer’s Disease-Like Pathology by Partially Rescuing Oxidative/Nitrosative Stress and Inflammation

**DOI:** 10.3390/ijms21051746

**Published:** 2020-03-04

**Authors:** Ana I. Duarte, Emanuel Candeias, Inês N. Alves, Débora Mena, Daniela F. Silva, Nuno J. Machado, Elisa J. Campos, Maria S. Santos, Catarina R. Oliveira, Paula I. Moreira

**Affiliations:** 1 CNC—Center for Neuroscience and Cell Biology, Rua Larga, Faculty of Medicine (Pólo 1, 1st Floor), University of Coimbra, 3004-504 Coimbra, Portugal; eu.emago@hotmail.com (E.C.); minalves09@gmail.com (I.N.A.); deboramena1996@gmail.com (D.M.); dani_malyk@hotmail.com (D.F.S.); nihonmeru@yahoo.co.uk (N.J.M.); mssantos@ci.uc.pt (M.S.S.); Catarina.n.oliveira@gmail.com (C.R.O.); 2 Institute for Interdisciplinary Research (IIIUC), University of Coimbra, Casa Costa Alemão-Pólo II, Rua D. Francisco de Lemos, 3030-789 Coimbra, Portugal; 3 Center for Innovative Biomedicine and Biotechnology (CIBB), Rua Larga, Faculty of Medicine (Pólo 1, 1st Floor), University of Coimbra, 3004-504 Coimbra, Portugal; elisajcampos@uc.pt; 4 Faculty of Medicine, University of Coimbra, 3004-504 Coimbra, Portugal; 5 Coimbra Institute for Clinical and Biomedical Research (iCBR), Faculty of Medicine, University of Coimbra, 3000-548 Coimbra, Portugal; 6 Life Sciences Department, University of Coimbra, Largo Marquês de Pombal, 3004-517 Coimbra, Portugal; 7 Institute of Biochemistry, Faculty of Medicine, University of Coimbra, 3000-548 Coimbra, Portugal; 8 Institute of Physiology, Faculty of Medicine, University of Coimbra, 3000-548 Coimbra, Portugal

**Keywords:** Alzheimer’s disease, brain protection, female sex, GLP-1 mimetics, liraglutide

## Abstract

Alzheimer’s disease (AD) is the most common form of dementia worldwide, being characterized by the deposition of senile plaques, neurofibrillary tangles (enriched in the amyloid beta (Aβ) peptide and hyperphosphorylated tau (p-tau), respectively) and memory loss. Aging, type 2 diabetes (T2D) and female sex (especially after menopause) are risk factors for AD, but their crosslinking mechanisms remain unclear. Most clinical trials targeting AD neuropathology failed and it remains incurable. However, evidence suggests that effective anti-T2D drugs, such as the GLP-1 mimetic and neuroprotector liraglutide, can be also efficient against AD. Thus, we aimed to study the benefits of a peripheral liraglutide treatment in AD female mice. We used blood and brain cortical lysates from 10-month-old 3xTg-AD female mice, treated for 28 days with liraglutide (0.2 mg/kg, once/day) to evaluate parameters affected in AD (e.g., Aβ and p-tau, motor and cognitive function, glucose metabolism, inflammation and oxidative/nitrosative stress). Despite the limited signs of cognitive changes in mature female mice, liraglutide only reduced their cortical Aβ_1–42_ levels. Liraglutide partially attenuated brain estradiol and GLP-1 and activated PKA levels, oxidative/nitrosative stress and inflammation in these AD female mice. Our results support the earlier use of liraglutide as a potential preventive/therapeutic agent against the accumulation of the first neuropathological features of AD in females.

## 1. Introduction

Alzheimer’s disease (AD) is the most common neurodegenerative disorder, neuropathologically characterized by the accumulation of senile plaques and neurofibrillary tangles (mainly composed of amyloid beta (Aβ) peptide and hyperphosphorylated tau protein (p-tau), respectively) [1,2]. Its most common clinical symptom is the progressive loss of memory [3]. 

Two-thirds of AD patients are women, >60% of them at menopause [4]. This renders female sex the major risk factor for sporadic AD after aging [5], with its pathophysiological action starting years to decades before the onset of clinical symptoms, most likely at midlife—the so-called prodromal or preclinical phase [6]. Indeed, studies showed that perimenopausal and menopausal women have a higher metabolic decline and Aβ levels, alongside a greater atrophy of grey and white matter relative to premenopausal women and age-matched men [7,8]. Although the involved mechanisms remain debatable, the hormonal fluctuations affecting women from midlife until advanced ages may render them more vulnerable to brain changes and AD [7,8,9,10]. In this respect, early changes in serum estrogen levels were correlated with cognitive impairment years later in aged women [11], and with cortical and hippocampal senile plaque formation and memory deficits in AD female mice [12,13,14,15,16,17]. This, together with the estimates that 2/3 of AD caregivers are women render them at the epicenter of this epidemic [7,8].

AD is closely connected with diabetes (particularly type 2 diabetes; T2D) and obesity—both considered risk factors for AD. Although evidence suggests that AD patients may be more prone to develop co-morbid diabetes or obesity, this remains debatable [18,19,20]. Nevertheless, the features shared by these pathologies (e.g., impaired insulin signaling, and brain glucose transport and metabolism, mitochondrial anomalies, redox imbalance, inflammation and cognitive deficits [20,21]), alongside the failure of most AD clinical trials, led to the hypothesis that antidiabetic drugs may have a therapeutic potential against AD. Among them, glucagon-like peptide-1 (GLP-1) analogs are highly promising, with a minimal hypoglycemic risk. Similar to endogenous GLP-1, they tightly regulate postprandial blood glucose-dependent insulin secretion, with a subsequent fall in glycemia [22]. GLP-1 is also ubiquitously expressed in the central nervous system (CNS), particularly in the hypothalamus, cortex, hippocampus, striatum, substantia nigra, brainstem and subventricular zone, where it may play a pivotal role [23]. Indeed, modulation of GLP-1 receptor protected against neurodegenerative events, neuronal death and cognitive decline [24,25]. Additionally, the GLP-1 mimetic liraglutide mitigated synaptic loss and neuropathology, and improved learning and memory in male AD mice [26,27]. Liraglutide also rescued hyperhomocysteinemia-induced AD pathology and memory deficits in rats [28]. Although the involved mechanisms remain unclear, liraglutide may recover brain insulin receptors (IR) and synapses after Aβ oligomer injection, ultimately improving memory function in mice and in non-human primates [29]. Liraglutide also hampered Aβ plaque formation [30], astrocyte- and microglia-mediated inflammation [31] and promoted neurogenesis and neuronal proliferation [32]. 

The lack of efficient AD-modifying therapies may result from studies performed in already symptomatic cohorts (with synaptic and neuronal deficits) and/or from the underestimation of sex differences in AD pathophysiology [33]. Moreover, most studies were performed in the hippocampus, despite the AD effects on wide areas of cerebral cortex [34,35] (including the frontal cortex) that underlie cognitive function and metabolic regulation [36]. Thus, there is an urgent need to uncover the role of female sex on brain cortical AD pathophysiology and progression, and to establish novel therapeutic strategies against the disease. These, by starting during the prodromal phase of AD, may efficiently prevent or delay its onset, or blunt its progression [33]. In this perspective, we aimed to evaluate the therapeutic benefits of a chronic (28-day) liraglutide treatment in mature female mice with AD-like pathology. Thus, we analyzed several brain parameters traditionally affected by AD, namely glucose metabolism, mitochondrial function/dynamics, inflammation, oxidative stress, neuropathological features and motor and cognitive behavior. 

As far as we know, only one study evaluated the effects of an 8-week liraglutide treatment in the 3xTg-AD mice, but in middle-aged (7–9 month-old) males [26]. This and our previous study in 11-month-old 3xTg-AD male mice [37] led us to use brain cortices from mature (10-month-old) 3xTg-AD female mice displaying AD-like pathology, treated with liraglutide for a shorter time (4 weeks). Our results suggest that, despite the limited signs of cognitive impairment in these mature female mice, liraglutide treatment only mitigated the increased accumulation of brain cortical Aβ_1–42_. The drug also partially normalized their brain estradiol, GLP-1 content and PKA, partially reducing their plasma and brain inflammatory and oxidative stress markers, possibly due to the stimulation of glucose 6-phosphate dehydrogenase (G6PDH) (and its downstream antioxidant properties) and mitochondrial dynamics. As far as we know, this study constitutes a first approach to the use of GLP-1 mimetics (namely liraglutide) to mitigate some of the earlier AD-like pathological features in females. Further studies are needed to reinforce the need for a more tailormade, sex/gender-based medicine.

## 2. Results

### 2.1. Effect of Liraglutide Treatment on Brain and Peripheral Features in Female Mice

The key neuropathological hallmarks of AD are the deposition of Aβ and hyperphosphorylated tau that occur early in disease pathology in brain areas such as the hippocampus and cortex, long before its clinical diagnosis that relies mostly on memory loss and, to a lower extent, in a few biomarkers [18,38]. However, the precise crosslinking mechanisms that occur across this timeframe remain debatable. 

**Figure 1:**
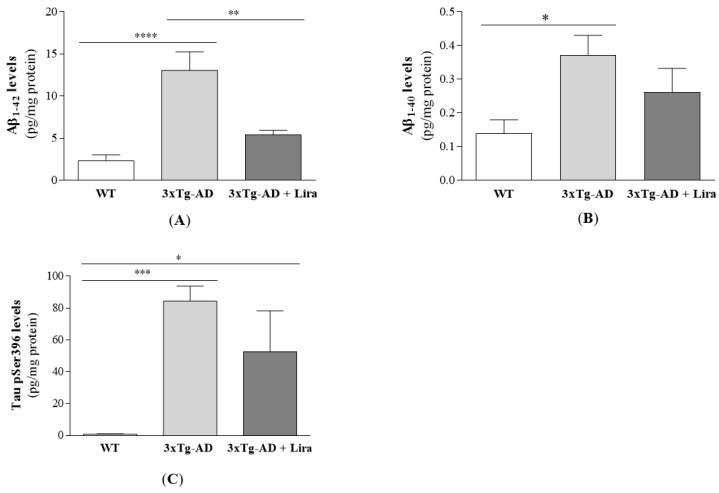
Effect of liraglutide on brain cortical AD-like hallmarks in 3xTg-AD female mice. Brain cortical Aβ_1–42_ (**A**), Aβ_1–40_ (**B**) and Tau pSer396 levels (**C**) were determined. Data are the mean ± SE from 4–6 mice/group. Statistical significance: * *p* < 0.05, ** *p* < 0.01, *** *p* < 0.001 or **** *p* < 0.0001, by the one-way ANOVA with the Bonferroni and Fisher LSD post-hoc tests for multiple comparisons.

**Figure 2:**
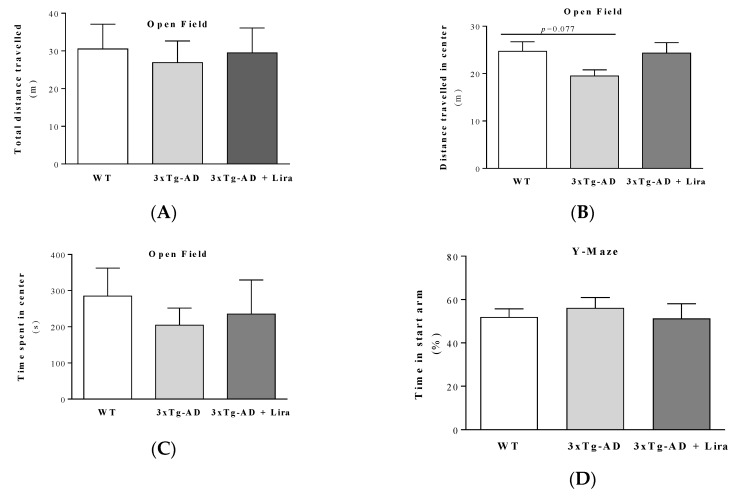

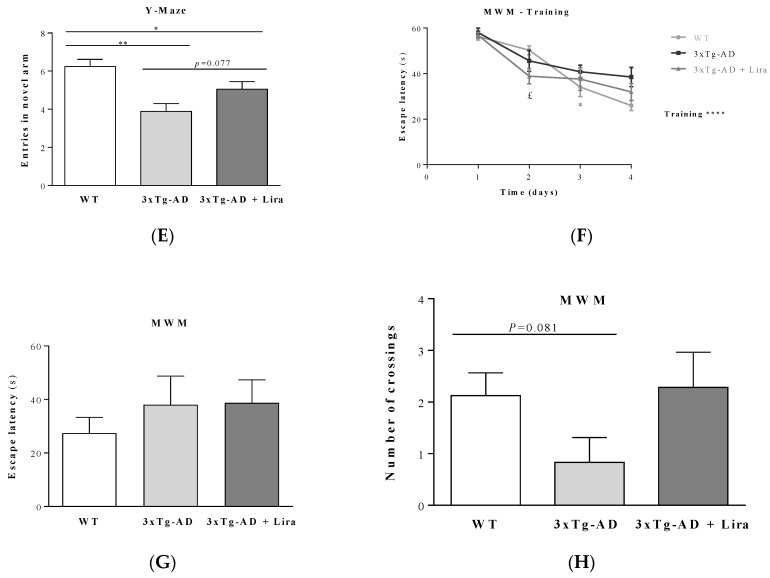
Effect of liraglutide on behavioral performance in female mice with early AD-like pathology. Total distance travelled (**A**), and distance travelled (**B**) and time spent in the center (**C**) of the open field area during the open field test; time spent in start arm during training (**D**) and number of entries into the novel arm during testing session (**E**) in the Y-maze test; escape latency across trainings days (**F**) and testing session (**G**), and the number of crossings during testing session (**H**) of the Morris Water Maze test were assessed. Data are the mean ± SE from 6–10 mice/group. Statistical significance: * *p* < 0.05 or ** *p* < 0.01, by the one-way ANOVA with the Fisher LSD post-hoc test for multiple comparisons (for a Gaussian distribution: A,B,D,E), or by the non-parametric Mann-Whitney test (for a non-Gaussian distribution: C,G,H). Regarding Figure 2F, statistical significance: * *p* < 0.05 in WT day 3 vs. WT day 2, ^£^*p* < 0.05 in 3xTg-AD + Lira day 2 vs. 3xTg-AD + Lira day 1, **** *p* < 0.0001 by two-way ANOVA, with the Tukey post-hoc test for multiple comparisons.

Similar to our previous study in 11-month-old 3xTg-AD male mice [37], here we observed a significant increase in brain Aβ_1–42_, Aβ_1–40_ and p-tau(Ser396) levels in 3xTg-AD female mice compared to WT ones. Liraglutide treatment only reduced brain Aβ_1–42_ levels (for Aβ_1–42_: *F*(2,14) = 15.206; *p* < 0.0001; for Aβ_1–40_: *F*(2,14) = 4.597; *p* = 0.029; for p-tau(Ser396): *F*(2,11) = 10.178; *p* = 0.003; Figure 1A–C). Despite this and our previous observations in mature 3xTg-AD male mice [37], our mature 3xTg-AD female mice only showed partial deficits in motor and cognitive performance compared to WT ones (Figure 2), as given by the slightly lower distance travelled in total (*F*(2,18) = 0.609; *p* = 0.554) and in the center of the open field arena (*F*(2,17) = 2.141; *p* = 0.148), and also by the time spent in its center (*Z* = −0.387, *p* = 0.755 for 3xTg-AD vs. WT mice; *Z* = −0.579, *p* = 0.613 for 3xTg-AD + Lira vs. WT mice; *Z* = −0.429, *p* = 0.731 for 3xTg-AD + Lira vs. 3xTg-AD mice), suggesting a thigmotaxic behavior that may be due to increased anxiety/fearfulness (Figure 2A–C). These were mirrored by their lower number of entries into the novel arm of the Y-maze (*F*(2,20) = 8.454; *p* = 0.002), despite no significant changes in the time spent in its start arm: *F*(2,21) = 0.259; *p* = 0.774) (Figure 2D,E), and the slightly reduced number of crossings of the Morris water maze (*Z* = −1.787, *p* = 0.081 for 3xTg-AD vs. WT mice; *Z* = −0.059, *p* = 0.955 for 3xTg-AD + Lira vs. WT mice; *Z* = −1.619, *p* = 0.138 for 3xTg-AD + Lira vs. 3xTg-AD mice; for escape latency: *Z* = −0.698, *p* = 0.536 for 3xTg-AD vs. WT mice; *Z* = −0.901, *p* = 0.408 for 3xTg-AD + Lira vs. WT mice; *Z* = −0.457, *p* = 0.710 for 3xTg-AD + Lira vs. 3xTg-AD mice) (Figure 2F–H), suggesting that the impairment in short-term spatial memory was not accompanied by significant changes in long-term spatial memory. Liraglutide administration only exerted limited benefits in these motor and cognitive deficits in mature 3xTg-AD female mice. 

These results suggest that our mature 3xTg-AD female mice model an early symptomatic stage of the disease, displaying early AD-like pathology with still limited signs of cognitive deficits. 

**Figure 3:**
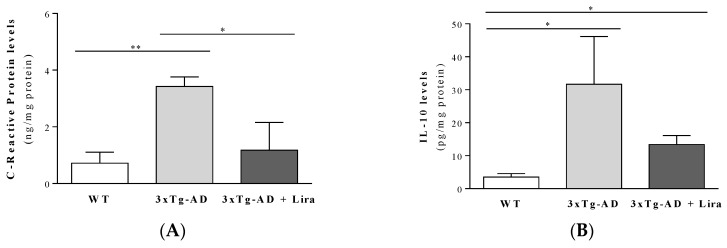
Effect of liraglutide on brain cortical inflammation markers in female mice with early AD-like pathology. Brain cortical C-Reactive Protein (**A**) and IL-10 (**B**) were determined. Data are the mean ± SE from 3–6 mice/group. Statistical significance: * *p* < 0.05 or ** *p* < 0.01, by the one-way ANOVA with the Fisher LSD or Games-Howell post-hoc tests for multiple comparisons.

Peripheral and brain inflammation constitutes another prominent feature of AD [39,40]. In line with this, we observed a massive increase in the pro-inflammatory CRP and IL-1β markers in plasma from the 3xTg-AD female mice, whereas the anti-inflammatory IL-10 was only slightly decreased (by 34%) compared to WT female mice (*F*(2,16) = 2.974; *p* = 0.08 for plasma CRP levels; for plasma IL-10 levels: *Z* = −0.857, *p* = 0.445 for 3xTg-AD vs. WT mice; for plasma IL-1β levels: *Z* = −2.882, *p* = 0.002 for 3xTg-AD vs. WT mice; Table 1). Liraglutide treatment tended to normalize the plasma inflammatory markers (for plasma IL-10 levels: *Z* = −0.319, *p* = 0.805 for 3xTg-AD + Lira vs. WT mice; *Z* = −1.286, *p* = 0.234 for 3xTg-AD + Lira vs. 3xTg-AD mice; for plasma IL-1β levels: *Z* = −2.00, *p* = 0.051 for 3xTg-AD + Lira vs. WT mice; *Z* = −1.143, *p* = 0.295 for 3xTg-AD + Lira vs. 3xTg-AD mice; Table 1). Similar to the well-described neuroinflammation markers in AD patients and animal models [41,42], the brains from 3xTg-AD female mice showed a significant increase in the pro-inflammatory CRP (*F*(2,11) = 9.337; *p* = 0.004) and in the anti-inflammatory cytokine IL-10 levels (*F*(2,14) = 2.447; *p* = 0.123) compared to WT female mice (Figure 3). Liraglutide treatment decreased their brain CRP and IL-10 levels (although the later was not statistically significant) (Figure 3). Unexpectedly, no significant alterations occurred in IL-1β levels in the brains from 3xTg-AD female mice (data not shown). These results further reinforce the notion that our 3xTg-AD female mice model an asymptomatic stage of the disease, displaying early AD-like neuropathology without substantial signs of cognitive deficits. This is further supported by the lack of significant alterations in brain weight (*F*(2,22) = 0.742; *p* = 0.868; Table 1) or in pre- and postsynaptic markers between experimental groups (data not shown).

Other feature of AD is body weight loss [43], whereas peripheral metabolic anomalies remain controversial [44]. Accordingly, our female mice with early AD-like pathology showed a 20% reduction in body weight that, nonetheless, was not recovered by liraglutide treatment (*F*(2,33) = 19.7; *p* < 0.0001; Table 1). Conversely, plasma estradiol levels were slightly increased (between 25–33%) in female mice with early AD-like pathology (treated or not with liraglutide) compared to WT mice (*F*(2,16) = 3.568, *p* = 0.052; Table 1). No significant alterations occurred in the peripheral glucose homeostasis markers occasional (*Z* = −0.139, *p* = 0.169 for 3xTg-AD vs. WT mice; *Z* = −0.129, *p* = 0.201 for 3xTg-AD + Lira vs. WT mice; *Z* = −0.129, *p* = 0.899 for 3xTg-AD + Lira vs. 3xTg-AD mice) and fasting glycemia (*F*(2,32) = 1.914, *p* = 0.153), HbA_1c_ (*F*(2,30) = 0.142, *p* = 0.868), plasma insulin (*Z* = −0.352, *p* = 0.756 for 3xTg-AD vs. WT mice; *Z* = −0.07, *p* = 0.973 for 3xTg-AD + Lira vs. WT mice; *Z* = −0.558, *p* = 0.606 for 3xTg-AD + Lira vs. 3xTg-AD mice), HOMA-IR (*Z* = −0.494, *p* = 0.654 for 3xTg-AD vs. WT mice; *Z* = −0.635, *p* = 0.557 for 3xTg-AD + Lira vs. WT mice; *Z* = −0.230, *p* = 0.847 for 3xTg-AD + Lira vs. 3xTg-AD mice) or HOMA-β (*Z* = −1.251, *p* = 0.236 for 3xTg-AD vs. WT mice; *Z* = −0.622, *p* = 0.573 for 3xTg-AD + Lira vs. WT mice; *Z* = −0.572, *p* = 0.604 for 3xTg-AD + Lira vs. 3xTg-AD mice) between experimental groups (Table 1). 

### 2.2. Liraglutide Partially Normalizes Brain Levels of Estradiol and GLP-1-Related Signaling in Female Mice with Early AD-Like Pathology

AD pathology has been associated with impaired levels and/or activity of hormones and signaling pathways [10,20,45]. Thus, we aimed to analyze the role of peripheral liraglutide treatment on brain estradiol and GLP-1 levels and downstream signaling in female mice with early AD-like pathology. 

Similar to the periphery, levels of brain estradiol and GLP-1 were increased in female mice with early AD-like pathology compared to WT ones (for brain GLP-1 levels: *F*(2,13) = 2.686; *p* = 0.106; for brain estradiol levels: *Z* = −2.191, *p* = 0.030 for 3xTg-AD vs. WT mice; Table 2). Liraglutide treatment tended to normalize both estradiol and GLP-1 levels (for brain estradiol levels: *Z* = −1.358, *p* = 0.222 for 3xTg-AD + Lira vs. WT mice; *Z* = −0.548, *p* = 0.662 for 3xTg-AD + Lira vs. 3xTg-AD mice; Table 2). Despite no significant alterations in brain insulin levels nor in IR, GLP-1R or activated Akt between cohorts (data not shown), female mice with early AD-like pathology had a massive decrease in brain active PKA kinase that tended to recover with liraglutide (*Z* = −2.562, *p* = 0.009 for 3xTg-AD vs. WT mice; *Z* = −0.548, *p* = 0.662 for 3xTg-AD + Lira vs. WT mice; *Z* = −0.913, *p* = 0.429 for 3xTg-AD + Lira vs. 3xTg-AD mice; Table 2). These results suggest an impairment in brain GLP-1R-mediated signaling in 3xTg-AD female mice that tended to be normalized by liraglutide administration (Table 2). 

### 2.3. Liraglutide Promotes Brain Glucose Metabolism via the Oxidative Branch of the Pentose Phosphate Pathway in Female Mice with Early AD-Like Pathology

Another feature of AD is the impairment in brain glucose transport and metabolism [46,47]. Therefore, we next evaluated the effect of liraglutide administration on brain cortical markers for glucose transport and downstream metabolism.

**Figure 4:**
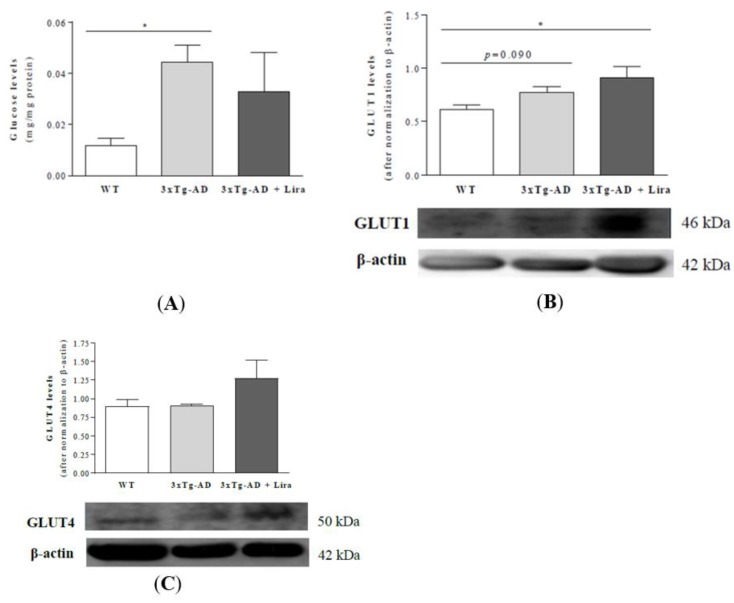
Effect of liraglutide on brain cortical glucose levels and transporters in mature female mice with early AD-like pathology. Brain cortical glucose (**A**), and GLUT1 (**B**) and GLUT4 protein levels (**C**) were evaluated and normalized to β-actin levels, and representative Western blotting images displayed. Data are the mean ± SE from 5–6 mice/group. Statistical significance: * *p* < 0.05, by the one-way ANOVA with the Fisher LSD or Games-Howell post-hoc tests for multiple comparisons.

Despite no significant alterations in GLUT4 and GLUT8 expression between experimental groups, brains from female mice with early AD-like pathology had higher glucose levels (*F*(2,14) = 2.433, *p* = 0.046 and slightly increased GLUT1 expression than WT mice (Figure 4A,B). Liraglutide treatment did not significantly affect brain GLUT1 and GLUT4 (an insulin-sensitive glucose transporter; *F*(2,13) = 4.491, *p* = 0.033) or glucose content in early AD-like female mice compared to 3xTg-AD female mice (Figure 4A–C). 

**Figure 5:**
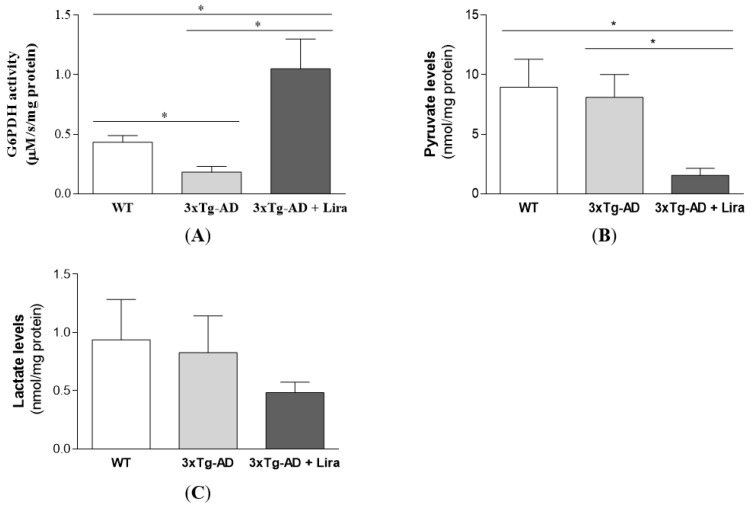
Effect of liraglutide on brain cortical glucose metabolism in female mice with early AD-like pathology. Brain cortical G6PDH activity (**A**), and pyruvate (**B**) and lactate levels (**C**) were determined. Data are the mean ± SE from 4–6 mice/group. Statistical significance: * *p* < 0.05, by the one-way ANOVA with the Fisher LSD post-hoc test for multiple comparisons (for a Gaussian distribution), or with the non-parametric Mann-Whitney test (for a non-Gaussian distribution: GAPDH activity and lactate levels).

Moreover, liraglutide abrogated the decrement in the activity of G6PDH (the limiting enzyme from the oxidative branch of the pentose phosphate pathway) in brains from female mice with early AD-like pathology (*Z* = −2.309, *p* = 0.029 for 3xTg-AD vs. WT mice; *Z* = −2.309, *p* = 0.029 for 3xTg-AD + Lira vs. WT mice; *Z* = −2.309, *p* = 0.029 for 3xTg-AD + Lira vs. 3xTg-AD mice; Figure 5A). Regarding glycolysis markers, liraglutide decreased brain pyruvate levels (*F*(2,15) = 5.210, *p* = 0.019) without significant changes in those of lactate in female mice with early AD-like pathology compared to the saline-treated ones (for lactate levels: *Z* = 0, *p* = 1 for 3xTg-AD vs. WT mice; *Z* = −0.838, *p* = 0.421 for 3xTg-AD + Lira vs. WT mice; *Z* = −0.548, *p* = 0.662 for 3xTg-AD + Lira vs. 3xTg-AD mice; Figure 5B,C). 

These results suggest that liraglutide-mediated stimulation of G6PDH may be beneficial against brain oxidative stress in female mice with early AD-like pathology.

### 2.4. Liraglutide Partially Rescues Brain Oxidative/Nitrosative Stress Markers in Female Mice with Early AD-Like Pathology

**Figure 6:**
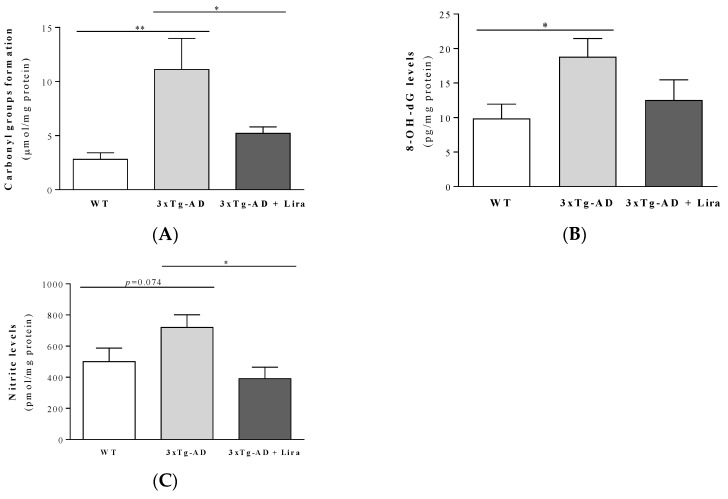
Effect of liraglutide on brain cortical oxidative and nitrosative stress markers in female mice with early AD-like pathology. Brain cortical carbonyl groups formation (**A**), 8-OH-dG (**B**) and nitrites levels (**C**) were determined. Data are the mean ± SE from 5–7 mice/group. Statistical significance: * *p* < 0.05 or ** *p* < 0.01, by the one-way ANOVA with the Fisher LSD post-hoc test for multiple comparisons.

From the above and since increased oxidative and nitrosative stress was demonstrated in both human and rodent AD brains (including the 3xTg-AD mice) [48,49], we next evaluated the effect of liraglutide on brain oxidative/nitrosative stress markers. Accordingly, brains from female mice with early AD-like pathology showed a slight increase in TBARS (by ~1.4-fold; *F*(2,13) = 2.819, *p* = 0.096; Supplementary Figure S1A) and nitrite levels (by ~1.4-fold; *F*(2,15) = 4.30, *p* = 0.033), and significantly higher carbonyl groups (by ~4-fold; *F*(2,14) = 5.755, *p* = 0.015) and 8-OHdG levels (by ~1.9-fold; *F*(2,15) = 3.559, *p* = 0.054) compared to WT mice (Figure 6A–C). Liraglutide tended to normalize the 8-OH-dG content (Figure 6B), while those of TBARS, carbonyl groups and nitrites were significantly reversed by the drug in female mice with early AD-like pathology (Figure 6A,C; Supplementary Figure S1A).

Recent evidence suggests that, besides its pivotal role in lysosomal-mediated autophagy, p62 may also be involved in oxidative defense, nutrient sensing and inflammation mechanisms [50]. Despite no significant alterations in brain p62 levels in female mice with early AD-like pathology, liraglutide treatment reduced its levels by 20% in these animals (*F*(2,15) = 4.424, *p* = 0.031; Supplementary Figure S1B).

These results suggest that peripheral treatment with liraglutide partially rescued brain oxidative stress markers in female mice with early AD-like pathology.

### 2.5. Liraglutide Partially Attenuates the Altered Mitochondrial Fission/Fusion Proteins in Female Mice with Early AD-Like Pathology

**Figure 7:**
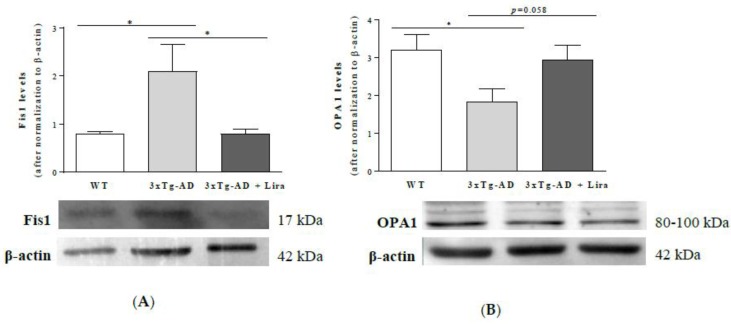
Effect of liraglutide on brain cortical mitochondrial fission/fusion markers in female mice with early AD-like pathology. Brain cortical Fis1 (**A**) and OPA1 protein levels (**B**) were determined and normalized to β-actin levels, and representative Western blotting images displayed. Data are the mean ± SE from 6 mice/group. Statistical significance: * *p* < 0.05, by the one-way ANOVA with the Fisher LSD post-hoc test for multiple comparisons.

Alongside the above-mentioned pathophysiological changes in AD, we previously showed alterations in brain mitochondrial dynamics [51]. Therefore, we aimed to study the role of liraglutide on brain markers for mitochondrial fission and fusion. We observed that liraglutide reversed the 2.6-fold increase in Fis1 levels in brains from female mice with early AD-like pathology (*F*(2,15) = 5.358, *p* = 0.018; Figure 7A), while the 1.8-fold lower OPA1 levels were only partially reversed upon liraglutide administration (by 1.6-fold) in female mice with early AD-like pathology (*F*(2,15) = 3.636, *p* = 0.052; Figure 7B). 

These results suggest that peripheral treatment with liraglutide partially attenuated the dysfunctional brain mitochondrial fission/fusion machinery in female mice with early AD-like pathology. 

## 3. Discussion

To the best of our knowledge, this study constitutes a first support to the use of GLP-1 mimetics (namely liraglutide) to mitigate some of the earlier AD-like pathological features in mature females. Contrary to our previous study in 11-month-old 3xTg-AD male mice that showed increased brain cortical and hippocampal Aβ levels and thigmotaxis, reduced exploratory activity, and deficits in learning and memory [37], in the present study the massive rise in brain cortical Aβ and p-tau content in 11-month-old 3xTg-AD female mice (in line with the *Amyloid Cascade Hypothesis*—the basis for this mouse model) was accompanied by less pronounced signs of cognitive alterations. Liraglutide treatment only attenuated their increased brain Aβ_1–42_ levels. This was accompanied by a slight reduction in their plasma and brain inflammatory markers upon liraglutide administration, which also tended to normalize estradiol and GLP-1 content, and PKA-mediated downstream signaling in female mice with early AD-like pathology. Interestingly, liraglutide partially mitigated their brain oxidative stress markers, possibly via the stimulation of G6PDH (and its downstream antioxidant properties) and by altering mitochondrial dynamics, ultimately rescuing the AD-like neuropathology in mature female mice.

Liraglutide administration attenuated memory deficits, Aβ plaques and oligomers, synaptic and tau pathology in APP/PS1 mice [27] and in non-human primates infused with Aβ oligomers into the lateral cerebral ventricle [29]. The drug also mitigated the cognitive deficits and cerebral p-tau in diabetic rodents [52,53]. However, others failed to observe a significant effect of chronic liraglutide treatment on cerebral Aβ plaque formation in two transgenic APP/PS1 mouse models with low and high grade of amyloidosis [54]. This suggested that distinct animal models for AD may display distinct sensitivities to liraglutide treatment [54]. Indeed, a recent study demonstrated that a 2-week administration of liraglutide decreased memory deficits, p-tau and Aβ overproduction, and increased dendritic spines’ density and synaptic proteins upon hyperhomocysteinemia [28]. In this respect, liraglutide injection for 4 weeks only mitigated the brain Aβ_1–42_ levels, without significantly affecting the Aβ_1–40_ or p-tau(Ser396) (a known intermediary phosphorylated residue in AD pathology [55,56]) in 3xTg-AD female mice with early AD-like pathology, which also presented less pronounced signs of motor, cognitive or synaptic defects (data not shown) (contrary to the previous observations of impaired motor activity and learning/memory in 3xTg-AD male mice [26,57]). This corroborates the slight delay in the onset of AD-like pathology in 3xTg-AD female mice described by Belfiore et al. [58], together with the notion of a sexual dimorphism in the susceptibility to AD neuropathology, cognitive dysfunction and changes in brain energy metabolism under neuropathological conditions [20,40,59,60] (including the persistently lower metabolic brain age in women across their life span compared to men [61]). Since Yan et al. [62] observed that peripheral 17β-estradiol treatment activates the estrogen receptor α and the downstream PI3K/Akt/Foxo1 signaling, recovering insulin sensitivity and glucose metabolism, one cannot exclude a role for the increased brain estradiol levels in this delay in AD-like neuropathology in 3xTg-AD female mice (as further discussed by Yang et al. [40]). Accordingly, Yang et al. [40] found that chronic 17β-estradiol administration to ovariectomized 3xTg-AD female mice recovered their spatial learning and memory, partially due to the recovery of PKA-CREB and downregulation of the p38-MAPK signaling. Hippocampal 17β-estradiol induced the release of glutamate from astrocytes, stimulating neuronal glutamate receptors, thereby modulating dendritic spine density and growth, and synapse formation and plasticity in developing and adult central nervous system [63,64]. Besides estradiol, the increased brain levels of GLP-1 in female mice with AD-like pathology may constitute an adaptive mechanism to delay the negative effects of less active PKA (its activation by hormones or neurotransmitters in multiple brain regions was shown to regulate feeding, energy expenditure and glucose homeostasis [65,66,67]). In line with this and with previous studies in AD patients and rodent models (including mature 3xTg-AD male mice) [37,68], our female mice with early AD-like pathology had lower body weight that, contrary to other animal models [69,70], was not recovered by liraglutide treatment. 

The delay in AD-like neuropathology in our female 3xTg-AD mice is further supported by their apparently unaltered peripheral glucose metabolism and insulin sensitivity, in contrast with previously studied mature 3xTg-AD male mice [57]. Although it is well-known that metabolic disorders (such as insulin resistance, T2D and/or obesity) increase the risk for AD [38,57,71,72,73,74], the opposite (i.e., AD-induced peripheral glucose dysmetabolism and insulin insensitivity) remains a matter of debate [75,76]. This does not invalidate the repurpose of anti-type 2 diabetes drugs to prevent or delay AD progression. Indeed, increasing evidence demonstrates the beneficial effects of, e.g., GLP-1 mimetics (including liraglutide) against AD [18,19,20]. Among them, we emphasize the liraglutide-induced recovery of brain glucose metabolism (whose changes may start before the onset of brain atrophy and neurodegeneration) [77,78,79,80,81,82,83]. Although the precise nature of such metabolic improvement remains unknown, evidence suggests a role for the recovered neurovascular unit (involving a NF-κB-induced balance between the vasoconstrictor endothelin-1 and the vasodilator endothelial nitric oxide synthase (eNOS)) [84,85] and the normalization of (cerebral) blood flow on the increment of GLUTs levels and/or function (their loss, particularly of those at the blood-brain barrier, like GLUT1 and, to a lesser extent, GLUT4, constitutes an early event in AD pathology) [86,87]. In addition, liraglutide-induced slowdown in brain glucose clearance may aid in the brain recovery of glucose uptake and/or metabolism (as our observations appear to partially confirm), ultimately, in improved cognitive performance [82,86,88,89,90,91,92]. However, others described that the tendentious increase in brain glucose metabolism induced by liraglutide upon AD was not accompanied by a rescue in cognitive function [19,93]. Hopefully, this apparent discrepancy will be clarified by a phase IIb trial involving the treatment of AD individuals with very mild dementia with liraglutide for 12-month (the ELAD trial) [77].

Oxidative/nitrosative stress and inflammation have been also widely demonstrated at the periphery [94,95,96,97] and in brains [49,98,99,100] of human subjects and rodent models of AD [40,48,101,102]. Several authors suggested that impaired redox status, Aβ deposition, neurofibrillary tangles and neuronal damage [103,104] play a key role in AD pathogenesis, most likely by activating microglia and inflammation-mediated neurotoxicity [105,106]. Accordingly, our female mice with early AD-like pathology had increased oxidative stress and serum and brain CRP and IL-1β levels. Indeed, high IL-1β levels occurred in AD patients and in mild cognitive impaired subjects [107,108], and activated microglia and astrocytes were recently correlated with the levels of hippocampal Aβ and p-tau, and the severity of AD pathology in 3xTg-AD mice [40]. This hippocampal Tau hyperphosphorylation may arise from an upregulation of the p-38-MAPK cascade in AD, while the downregulation of cAMP-PKA-CREB signaling (as partially observed in Table 2) may impair synaptic plasticity and memory formation [40]. Importantly, the role of the anti-inflammatory cytokine IL-10 in AD brain remains controversial, since recent studies in APP mice suggested that it may inhibit microglial Aβ clearance, promoting Aβ plaque generation and cognitive impairment (rather than delaying AD progression) [109]. Furthermore, brain immunity was improved in IL-10-deficient APP mice that also showed lower cerebral amyloidosis [110]. Hence, the increased brain IL-10 content in female mice with early AD-like pathology appears to precede their typical behavioral deficits, possibly exacerbating the brain damage elicited by IL-1β, CRP and oxidative/nitrosative stress and allowing AD progression. In line with previous studies [27,111], liraglutide partially mitigated brain oxidative stress and inflammation markers in female mice with early AD-like pathology. 

Similar to liraglutide’s anti-inflammatory mechanisms, those underlying its anti-oxidative stress properties remain poorly understood. These may involve the activation of Akt and eNOS, with the subsequent stimulation of antioxidant defenses (e.g., glutathione, catalase, superoxide dismutase) and reduction of reactive oxygen species (ROS) formation, as observed in ischemic stroke [112,113]. Despite no significant alterations in active Akt in our conditions, one cannot exclude the involvement of the parallel MAPK/ERK signaling cascade [112], known to mediate its antioxidant, anti-inflammatory, anti-apoptotic and pro-cognition roles [114,115,116,117,118,119,120,121], as well as its benefits in AD symptoms and features [77]. Liraglutide-mediated NF-κB inhibition and Sirt1 may also recover mitochondrial membrane integrity and complex I activity, improving mitochondrial function (as reported in epilepsy, ischemia or toxin exposure) [18,112,122,123,124,125,126,127,128,129], and further protecting against oxidative stress [112,118,130,131], which may also rely on the inhibition of myeloperoxidase (via Nrf2/heme oxygenase-1 downregulation of NADPH oxidase or PKCα membrane translocation, as reported in diabetic and stroke brain) [132]. Importantly, the lower G6PDH activity (a pivotal enzyme from the oxidative branch of the pentose phosphate pathway also involved in the regulation of nicotinamide adenine dinucleotide phosphate (NADPH) and of the key antioxidant reduced glutathione, GSH) observed in brains from female mice with early AD-like pathology further support an increased oxidative stress, in agreement with the G6PDH inhibition in *postmortem* hippocampal regions [133] and prefrontal cortex synaptosomes [134] from AD human subjects. The liraglutide-mediated increase in G6PDH activity and decreased pyruvate levels in mature female mice with early AD-like pathology suggest that its antioxidant effects may involve the stimulation of the oxidative branch of the pentose phosphate pathway (rather than glycolysis) and/or a decrement in p62 levels. Since the liraglutide-induced changes in this stress-inducible protein were not accompanied by alterations in other autophagy markers (p62 is mostly known as a cargo receptor for the lysosomal-mediated autophagy degradation of detrimental and unnecessary components), we hypothesize that p62 may alternatively account for liraglutide’s anti-oxidative stress or anti-inflammatory properties. Indeed, p62 was recently associated with Nrf2, mTORC1 and NF-κB signaling pathways and their role in oxidative stress, nutrient sensing and inflammation [50]. Besides the liraglutide’s anti-inflammatory mechanisms discussed above, NF-κB inhibition was also found to reduce TNFα, IL-1β and IL-6 levels, and activated microglia and astrocytes [25,123,135,136,137,138], while the downregulation of JNK and phosphorylated p38, and the consequent inhibition of caspases-8 and -3, may account for its anti-apoptotic actions [112,115,139,140].

The increased Fis1 and decreased OPA1 levels in female mice with early AD-like pathology suggest a dysregulation in brain mitochondrial fission/fusion machinery, namely the promotion of fission and the impairment of fusion processes [141,142,143], respectively. OPA1 at the mitochondrial inner membrane is also involved, e.g., in the maintenance of mitochondrial respiratory chain and membrane potential [144], cristae organization, mitochondrial DNA and apoptosis regulation [145,146,147], whereas Fis1 can also regulate the size and distribution of mitochondria in response to the local demand for ATP or calcium [148]. Hence, changes in brain OPA1 and Fis1 levels in female mice with early AD-like pathology may elicit alternative damaging mechanisms that were partially reversed by liraglutide.

Although not studied herein, the anti-amyloidogenic/tauogenic effects of liraglutide may also rely on the PI3-K/MAPK/cAMP/PKA-mediated activation of brain insulin degrading enzyme (IDE) and/or the upregulation of Aβ transporters to promote Aβ trafficking and proteolytic degradation [149,150,151,152,153,154]; on the inactive caspase-3-mediated blunt of neurofibrillary tangle formation [112,155,156,157]; on the regulation of brain neurotransmission (e.g., GABAergic and glutamatergic) [158,159,160,161,162,163], thus promoting synaptic plasticity; on the improvement of axonal sprouting and neurite outgrowth [130,164,165,166]; and/or on increased neurogenesis [21,30,136,167,168,169], ultimately contributing to (AD) brain repair and cognitive function [130,170,171,172]. Finally, in spite of the apparent lack of changes in the present study, we cannot underestimate the indirect peripheral effects of liraglutide in restoring insulin action and glucose homeostasis, as well as in blood pressure, body weight and lipid profiles [25,30,160,173,174,175,176,177,178,179,180,181].

Altogether, our results constitute a first approach to disentangle the complex puzzle underlying the use of the GLP-1 mimetic liraglutide as a potential preventive/therapeutic agent against some of the earlier AD-like pathological signs in female mice. Although further studies are needed (particularly in rodent models displaying risk factors for sporadic AD, including aging or diabetes), the different patterns in AD-related pathology between males and females and their response to medicines also reinforce the need for a more tailormade, sex/gender-based medicine.

## 4. Material and Methods

### 4.1. Materials

Bovine serum albumin (BSA), phenylmethylsulfonyl fluoride (PMSF), dithiothreitol (DTT), Tween 20, thiobarbituric acid (TBA) and mouse monoclonal β-actin (#A5441) antibody were obtained from Sigma-Aldrich (St. Louis, MO, USA). Polyvinylidene difluoride (PVDF) Immobilon-P membranes and rabbit polyclonal glucose transporter 1 (GLUT1, #CBL242) antibody were obtained from Millipore (Billerica, MA, USA). Mouse monoclonal GLUT4 antibody (#2213S) was obtained from Cell Signaling (Leiden, The Netherlands). Mouse monoclonal OPA1 antibody (#612607) was obtained from BD Biosciences (Oeiras, Portugal). Rabbit polyclonal mitochondrial fission 1 protein (Fis1, #NB100-56646) antibody was obtained from Novus Biologicals (Abingdon, United Kingdom). Anti-mouse and anti-rabbit secondary antibodies (#RPN5781 and #RPN5783), and enhanced chemifluorescence (ECF) reagent were purchased from Amersham Biosciences (Little Chalfont, UK). Rat Insulin Enzyme Immunoassay kit (#A05105) was purchased from SPI-BIO, Bertin Pharma (Montigny le Bretonneux, France). Estradiol EIA kit (#582251) and 8-hydroxy-2-deoxy guanosine EIA (#589320) kit were purchased from Cayman Chemical (Ann Arbor, USA). QuantiChrom Glucose Assay kit (#DIGL-100) was purchased from BioAssay Systems (Hayward, CA, USA). Rat Amyloid Beta Peptide 1–42 ELISA kit (#LTI KMB3441) was purchased from EIAab Science Co. (Wuhan, China). Mouse β Amyloid 1–40 ELISA kit (#LTI KMB3481) and Tau [pS396] Human ELISA Kit (#LTI KHB7031) were purchased from Invitrogen (Camarillo, CA, USA). Trichloroacetic acid (TCA) was purchased from Calbiochem (Merck KGaA, Darmstadt, Germany). Rat GLP-1 ELISA Kit (#E-EL-R0059) was purchased from Elabscience (Wuhan, Hubei, China). Rat C-Reactive Protein (CRP) ELISA Kit (#88-7501-28), Rat interleukin (IL)-1β Platinum ELISA kit (#BMS630) and Rat IL-10 Platinum ELISA kit (#BMS629) were purchased from eBioscience (Vienna, Austria). Protein kinase A (PKA) kinase activity kit (#ADI-EKS-390A) was purchased from Enzo Life Sciences, Grupo Taper SA (Sintra, Portugal). All other chemicals used were of the highest grade of purity commercially available.

### 4.2. Animal Housing and Treatment

Following EU and Portuguese legislation (Directive 2010/63/EU; DL113/2013, August 7th) and ARRIVE guidelines [182], 10 month-old WT (control) and 3xTg-AD female mice (a genetic model for AD that develops an age-related progressive neuropathological phenotype) [57] were used upon ethical approval by the Animal Welfare Committee of the Center for Neuroscience and Cell Biology and Faculty of Medicine, University of Coimbra (Project ORBEA_61_2013/24072013). Following the “3Rs” Reduction principle established by FELASA, in a first approach we used the brain cortical GLP-1 levels of saline-treated WT and 3xTg-AD female mice (Table 2) to estimate the number of animals required for this study. Briefly, by using the Wilcoxon-Mann-Whitney test applied to their independent means and standard deviations on the G-Power software [183], an alpha error of 0.05 and a power of 80%, we estimated that a total of six mice should be used for the overall study. In line with this and aiming to increase the power of our hypothesis, we used a minimum of four mice per parameter.

Mice were maintained at our animal colony (Animal Research Center, University of Coimbra) in static microisolator cages (3–4 mice/cage) with a filter top and bedding and nesting materials, under controlled light (12h day/night cycle) and humidity (45–65%) and *ad libitum* standard hard pellets chow and sterilized and acidified water (pH 2.5–3). Signs of distress were carefully monitored. Mice were randomly divided into three experimental groups: in the first one, 14 3xTg-AD female mice were daily, subcutaneously (s.c.) injected with liraglutide (0.2mg/kg), for 28 days, whereas the remaining two groups (10 wild type and 12 3xTg-AD mice; mice with AD-like pathology were subjected to random assignments) received saline injection (0.9% sterile NaCl). Although not expected, a rapid decrease in body weight >15–20% was defined as a humane endpoint for the study. 

### 4.3. Body and Brain Weight 

Body weight was monitored once/week throughout the study. Immediately before euthanasia, animals were also weighed. After euthanasia, brains were immediately removed and weighed. Results were expressed as body weight or brain weight (g). 

### 4.4. Collection of Peripheral Blood and Routine Biochemical Analyses

Mice were fasted for ~6h (starting early in the morning) and immediately after their euthanasia blood was immediately collected directly from the heart by transcardial punction to commercially-available blood collection tubes containing EDTA (Vacuette^®^ K3E/EDTA3K; Greiner Bio One, Kremsmünster, Austria) to isolate plasma (as detailed below). One drop of blood was used to determine fasting or occasional blood glucose levels by the glucose oxidase reaction, using a glucometer (Glucometer-Elite, Bayer SA, Portugal) and compatible stripes. Results were expressed as mg glucose/dL blood. 

Blood glycated hemoglobin (HbA_1c_) was measured with the Multi-Test HbA1c (A1C Now+, Bayer SA, Portugal) and results expressed as %. The remaining blood was centrifuged at 572× *g* for 10 min, at 4 °C, in a Sigma 2–16 PK centrifuge. The resulting plasma was used to determine fasting insulin levels through the Insulin Enzyme Immunoassay kit, according to the manufacturer’s instructions. Absorbance was read at 405 nm in a SpectraMax Plus 384 multiplate reader, when maximum binding (B_0_) wells reached 0.2–0.8 arbitrary units (a.u.) Results were expressed as ng/mL plasma. 

Plasma estradiol levels were measured by the Estradiol EIA kit, according to the manufacturer’s instructions. Absorbance was read at 450 nm, in a SpectraMax Plus 384 multiplate reader. Results were expressed as pg/mL plasma.

### 4.5. Isolation and Preparation of Brain Cortical Homogenates

After euthanasia, brains were immediately removed and cortices dissected and snap-frozen for further studies. Brain cortices were then homogenized at 0–4 °C in lysis buffer, containing (in mM): 25 HEPES, 2 MgCl_2_, 1 EDTA, 1 EGTA, pH 7.4, supplemented with 2 mM DTT, 100 µM PMSF and commercial protease and phosphatase inhibitors cocktails. The crude homogenate was centrifuged at 17,968× *g* for 10 min, at 4 °C in a Sigma 2–16K centrifuge to remove the nuclei, and the resulting supernatant was collected. Pellet was further resuspended in supplemented buffered solution and centrifuged again at 17,968× *g* for 10 min, at 4 °C. The supernatant was added to the previously obtained one and protein content determined by the Bio-Rad Protein Assay, according to the manufacturer’s instructions. 

### 4.6. Evaluation of AD Pathological Hallmarks

Brain cortical Aβ_1–42_ levels were determined in 10 µL brain cortical homogenates by the Amyloid Beta Peptide 1–42 ELISA kit, according to the manufacturer’s instructions. Absorbance was determined at 450 nm, in a SpectraMax Plus 384 multiplate reader. Results were expressed as pg/mg protein. 

Brain cortical Aβ_1–40_ levels were determined in 10 µL of brain cortical homogenates by the β-Amyloid 1–40 ELISA kit, according to the manufacturer’s instructions. Absorbance was determined at 450 nm, in a SpectraMax Plus 384 multiplate reader. Results were expressed as pg/mg protein. 

Brain cortical levels of p-tau protein at the serine 396 residue (Tau pSer396) were determined in 10 µL of brain cortical homogenates by the Tau [pS396] Human ELISA Kit, according to the manufacturer’s instructions. Absorbance was read at 450 nm in a SpectraMax Plus 384 multiplate reader. Results were expressed as pg/mg protein. 

### 4.7. Behavioral Analyses

At the end of treatment, mice were transported in their home cages to the behavioral testing room and allowed to acclimate to the room for at least 2h prior to each test. Behavioral tests were performed in consecutive days, by experienced observers blind to the experimental conditions.

#### 4.7.1. Open Field Behavior Test 

Open field behavior testing allows the assessment of the locomotor and behavioral activity in rodents [184]. Motor activity was evaluated during night cycle in an open field squared arena with grey open-topped boxes (50 cm wide × 50 cm deep × 40 cm high), using the Stoelting ANY-MAZE video tracking system (Stoelting Co., Wood Dale, IL, USA), detecting position of the animal’s head. Mice were placed individually in the corner of the open field arena and were recorded for a 30-min period. Data were collected every 5 min.

#### 4.7.2. Y-maze Behavior Test 

Short-term spatial memory was evaluated using the modified Y-maze test, based on the innate preference of animals to explore areas that have not been previously explored [185]. Briefly, using a Y-shaped plexiglass apparatus consisting of three arms (18 cm long, 6 cm wide and 6 cm high) separated by equal angles, mice were subjected to a training session whereby they freely explored two arms (Start and Other) for 8 min, while the third one (Novel) was blocked [186,187,188]. After a 120-min inter-trial interval, mice were subjected to the test session, after the removal of the wall that blocked the Novel arm and its opening for free exploration of the three arms for 8 min. Memory performance was given by the percentage of time spent in the novel arm over the time spent exploring all arms.

#### 4.7.3. Morris Water Maze Test

Spatial memory was assessed by the Morris water maze (MWM) test, as described by Morris et al. [189], with slight modifications [185]. Briefly, tests were performed in a circular swimming pool made of grey-painted fiberglass, 1.2 m inside diameter, 0.8 m high, which was filled to a depth of 0.6 m with water maintained a 23 ± 2 °C. The target platform (10 × 10 cm^2^) of transparent acrylic resin was submerged 1–1.5 cm beneath the water surface and it was cued by a 7-cm diameter white ball attached to the top of the platform and protruding above the water. Starting points were marked on the outside of the pool as north (N), south (S), east (E) and west (W). Four distant cues (55 × 55 cm^2^) were placed 30 cm above the upper edge of the water tank and the position of each symbol marked the midpoint of the perimeter of a quadrant (circle = NE quadrant, square = SE quadrant, cross = SW quadrant and diamond = NW quadrant). A monitor and a video-recording system were installed in an adjacent room. 

Mice were submitted to a cued version of the water maze [190], consisting of four training days and four consecutive trials per day, during which the animals were left in the tank facing the wall and were then allowed to swim freely to the submerged platform placed in the center of one of the four imaginary quadrants of the tank. The initial position in which the animal was left in the tank was one of the four vertices of the imaginary quadrants of the tank, by the following order: north, south, east and west. If the mouse did not find the platform during a period of 60 s, it was gently guided to it. After the animal had escaped to the platform, it remained on it for 10 s and was then removed from the tank for 20 s before being placed in the next random initial position. Test session (day five) consisted of a single trial, in which the platform was removed and each mouse was allowed to swim for 60 s in the maze. The experiments were recorded and the scores for latency of escape from the starting point to the platform and swimming speed were later measured with the ANY-MAZE™ video tracking system.

### 4.8. Evaluation of Inflammation Markers

Inflammation markers were evaluated in plasma and brain cortical homogenates, by using the C-Reactive Protein (CRP) ELISA Kit, IL-10 Platinum ELISA kit and IL-1β Platinum ELISA kit, according to the manufacturer’s instructions. Briefly, 7.5 µL of plasma and 5 µL of each brain cortical homogenate were used to determine CRP levels, whereas 10 µL of plasma and each brain cortical homogenate were used for IL-10 and IL-1β levels. Absorbance was read at 450 nm, in a SpectraMax Plus 384 multiplate reader. Results were expressed as ng/mL plasma and ng/mg protein for CRP, and as pg/mL plasma and pg/mg protein for IL-1β and IL-10.

### 4.9. Evaluation of Brain Cortical Hormones’ Levels

Brain cortical estradiol levels were measured in 10 µL of each sample (with the remaining volumes decreased to half) by using the Estradiol EIA kit, according to the manufacturer’s instructions. Absorbance was determined by a SpectraMax Plus 384 multiplate reader, at 450 nm. Results were expressed as pg/mg protein.

Brain cortical GLP-1 levels were measured in 20μL of each sample (working dilution of 1:5) by the Rat GLP-1 ELISA Kit. Absorbance was determined at 450 nm, in a SpectraMax Plus 384 multiplate reader. Results were expressed as pg/mg protein.

### 4.10. Assessment of Brain Cortical PKA Activity

Active PKA kinase was determined in 5 µL of each sample (working dilution of 1:6) by the PKA kinase activity kit. Absorbance was determined at 450 nm, in a SpectraMax Plus 384 multiplate reader. Results were expressed as ng/mg protein.

### 4.11. Assessment of Brain Cortical Glucose Levels

Brain cortical glucose levels were determined by the QuantiChromTM Glucose Assay kit, according to the manufacturer’s instructions, in 5 µL of each brain cortical homogenate. Absorbance was read at 630 nm, in a SpectraMax Plus 384 multiplate reader. Results were expressed as mg/mg protein.

### 4.12. Determination of Brain Markers for Glycolysis and Pentose Phosphate Pathway

Glycolytic metabolism and pentose phosphate pathways were given by the activity of the pentose phosphate pathway enzyme G6PDH, and by the levels of pyruvate and lactate in mouse brain cortical lysates.

Pentose phosphate pathway was given by the activity of G6PDH, that catalyzes the formation of 6-phosphogluconolactone from G6P, at the expense of NADP^+^, according to a previously described method [191]. Briefly, 5 µL of each brain cortical lysate were incubated in a reaction buffer containing 50 mM Tris-HCl (pH 7.5) and supplemented with 50 μM MgCl_2_ and 7.2 μM NADP^+^. Absorbance was read at 340nm, at 37 °C, during 2 min, with readings of 20 s intervals, in a SpectraMax Plus 384 microplate reader. Then, the reaction was initiated by the addition of 0.5 mM G6P, and the absorbance continuously read for 150 s, with 20 s intervals. G6PDH activity was calculated using a ε340nm = 6220 M^−1^cm^−1^. Results were expressed as µM/s/mg protein. 

Pyruvate levels were determined by the Pyruvate Colorimetric/Fluorometric assay kit, according to the manufacturer’s instructions, in 5 µL of brain cortical lysate (working dilution 1:10). Absorbance was read at 570 nm, in a SpectraMax Plus 384 microplate reader. Results were expressed as nmol/mg protein. 

Lactate levels were determined by the Lactate Colorimetric/Fluorometric assay kit, according to the manufacturer’s instructions, in 5 µL of each brain cortical homogenate (working dilution 1:10). Absorbance was read at 570 nm, in a SpectraMax Plus 384 microplate reader. Results were expressed as nmol/mg protein. 

### 4.13. Evaluation of Oxidative/Nitrosative Stress Markers

Carbonyl groups were determined according to Fagan et al. [192], with slight modifications. Briefly, 5 µL of each brain cortical homogenate were dissolved in 71 µL TCA 20%, and centrifuged at 9167× *g*, for 3 min, in a Sigma 2–16K centrifuge. The pellet obtained was incubated for 1h, at room temperature, in 35 µL DNPH 10 mM (freshly prepared in 2M HCl) protected from light and with vortex agitation every 10min. Then, 35 µL TCA 20% were added and the mixture was centrifuged at 11,092× *g*, for 3 min. The resulting pellet was mixed with 71 µL ethanol:ethyl acetate (1:1, *v/v*), and centrifuged again at 9167× *g*, for 3 min. Then, the pellet was incubated in 64.3 µL guanidine 6M (prepared in PBS, pH 6.5), for 15 min, at 37 °C, and centrifuged at 9167× *g* for 3 min. For all samples, a blank was prepared, which was incubated with HCl 2M instead of DNPH. Carbonyl content was calculated from the maximum absorbance, at 360 nm, measured in a SpectraMax Plus 384 multiplate reader, and an ε_360nm_ = 22 × 10^3^ M^−1^cm^−1^. The results were expressed as µmol/mg protein.

Levels of the DNA oxidation marker 8-hydroxy-2-deoxy guanosine (8-OH-dG) were determined in 10 µL of brain cortical homogenates by the 8-OH-dG EIA kit (Cayman Chemical Co.), according to the manufacturer’s instructions. Absorbance was read at 405 nm, in a SpectraMax Plus 384 multiplate reader. Results were expressed as pg/mg protein. 

Nitrite levels were indirectly given by the NO^•^ production upon the reaction with Griess reagent, according to Green et al. [193]. Briefly, 100 µg of each brain cortical homogenate were diluted in 100 µL phosphate buffer and incubated, for 10 min, in 100 µL Griess reagent (containing 1% sulfanilamide in 2.5% phosphoric acid, plus 0.1% *n*-(1-naphthyl) ethylenediamine dihydrochloride), protected from light. Absorbance was read at 550nm, in a SpectraMax Plus 384 multiplate reader. Nitrite content was calculated using a standard curve of sodium nitrite. Results were expressed as pmol/mg protein.

### 4.14. Western Blot Analyses

Samples containing denatured brain cortical homogenates (50 µg per lane) were subjected to sodium dodecyl sulfate (SDS)/polyacrylamide gel electrophoresis (SDS/PAGE) (8–15%) and transferred onto polyvinyl difluoride (PVDF) membranes. Then, membranes were blocked for 1h at room temperature in Tris-buffered saline (TBS, pH 7.4) plus 1% or 5% BSA and 0.05% Tween 20. Membranes were then incubated overnight at 4ºC with rabbit GLUT1 (1:1000), mouse GLUT4 (1:1000), rabbit Fis1 (1:750) and mouse OPA1 (1:1000) primary antibodies. Membranes were then incubated with the respective anti-rabbit or -mouse secondary IgG antibodies (1:10,000), for 2h, at room temperature, and developed using ECF. Immunoreactive bands were visualized by the VersaDoc Imaging System (Bio-Rad, Hercules, CA, USA). Fluorescence signal was analyzed using the QuantityOne software and the results given as INT/mm^2^. Of note, membranes were then reprobed with the corresponding mouse β-actin (1:5000) primary antibody. Results were presented as the ratio between total protein vs. β-actin.

### 4.15. Statistical Analysis

Authors performed the statistical analysis using SPSS version 24.0 (IBM Corp., Armonk, NY, USA). The extreme outliers were discarded, based on the 3× IQR criterion. The Shapiro-Wilk test was used to assess the normality of data (*p* > 0.05), since the number of mice/group were considered small (i.e., *n* < 50). The normally distributed data were evaluated concerning the homogeneity of variance, using the Levene’s test (*p* > 0.05). For data with a Gaussian distribution, a parametric one-way analysis of variance (ANOVA) was performed to determine whether there were significant overall differences (*p* < 0.05) between the mean of more than two groups. To determine which groups differed from the rest (*p* < 0.05), the Fisher’s Least Significant Difference (LSD), Bonferroni or the Games-Howell *post-hoc* tests were used. For data with a non-Gaussian distribution, a non-parametric Mann-Whitney test was used (*p* < 0.05). In this study, the groups analyzed were the brain cortical homogenates, blood or plasma from mature female WT mice, 3xTg-AD and 3xTg-AD + Liraglutide mice. Statistical significance was defined as *p* < 0.05. 

Graphic artwork was obtained using the GraphPad Prism 6.0 software (GraphPad Software, San Diego, CA, USA). Data were presented as mean + SE of the indicated number of mice/group, run in duplicate. 

## Figures and Tables

**Table 1:** Effect of liraglutide administration on peripheral features of female mice with early AD-like pathology.

	WT	3xTg-AD	3xTg-AD + Lira
**Body weight** (g)	29.1 ± 1.2 (*n* = 10) (95% CI: 26.3–31.8)	23.3 ± 0.6 **** (*n* = 12) (95% CI: 22.1–24.6)	23.3 ± 0.4 **** (*n* = 14) (95% CI: 22.6–24.1)
**Brain weight** (g)	0.5 ± 0.01 (*n* = 7) (95% CI: 0.45–0.51)	0.4 ± 0.03 (*n* = 8) (95% CI: 0.36–0.52)	0.5 ± 0.03 (*n* = 10) (95% CI: 0.42–0.54)
**HbA_1c_** (%)	4.3 ± 0.2 (*n* = 10) (95% CI: 3.74–4.84)	4.4 ± 0.1 (*n* = 11) (95% CI: 4.17–4.65)	4.4 ± 0.1 (*n* = 12) (95% CI: 4.13–4.57)
**Occasional glycemia** (mg glucose/dL blood)	132.8 ± 3.3 (*n* = 9) (95% CI: 125.2–140.3)	121.2 ± 7.3 (*n* = 12) (95% CI: 105.2–137.2)	128.1 ± 10.5 (*n* = 14) (95% CI: 105.6–150.7)
**Fasting glycemia** (mg glucose/dL blood)	126.4 ± 4.7 (*n* = 9) (95% CI: 115.6–137.7)	110.3 ± 8.2 (*n* = 12) (95% CI: 92.4–128.3)	127.6 ± 6.5 *p* = 0.073 (*n* = 14) (95% CI: 113.7–141.6)
**Fasting insulin levels** (ng/mL plasma)	3.5 ± 1.5 (*n* = 10) (95% CI: 0.07–6.97)	2.5 ± 0.8 (*n* = 11) (95% CI: 0.72–4.23)	1.3 ± 0.4 (*n* = 11) (95% CI: 0.56–2.13)
**HOMA-IR**	30.2 ± 13.1 (*n* = 10) (95% CI: 0.7–59.8)	15.2 ± 5.0 (*n* = 11) (95% CI: 4.05–26.37)	11.3 ± 3.1 (*n* = 11) (95% CI: 4.37–18.26)
**HOMA-β**	217.5 ± 124.23 (*n* = 8) (95% CI: −76.25–511.26)	262.1 ± 93.01 (*n* = 9) (95% CI: 47.60–476.5)	170 ± 43.33 (*n* = 10) (95% CI: 72–268)
**Estradiol levels** (pg/mL plasma)	184.1 ± 15.1 (*n* = 7) (95% CI: 147.2–220.9)	230.8 ± 24.3 *p* = 0.07 (*n* = 6) (95% CI: 168.3–293.3)	244.9 ± 9.5 *p* = 0.023 (*n* = 6) (95% CI: 220.3–269.4)
**C-Reactive Protein levels** (ng/mL plasma)	31.9 ± 6.1 (*n* = 6) (95% CI: 16.25–47.51)	74.3 ± 17.6 * (*n* = 6) (95% CI: 29.10–119.4)	60.8 ± 10.7 (*n* = 7) (95% CI: 34.53–86.98)
**IL-10 levels** (pg/mL plasma)	551.5 ± 134.6 (*n* = 7) (95% CI: 222.1–880.9)	364.6 ± 81.6 (*n* = 6) (95% CI: 154.8–574.3)	494.3 ± 54.5 (*n* = 7) (95% CI: 361.1–627.6)
**IL-1β levels** (pg/mL plasma)	43.2 ± 12.3 (*n* = 6) (95% CI: 11.66–74.66)	821.6 ± 400.7 ** (*n* = 6) (95% CI: −208.3–1852)	355.4 ± 159.3 *p* = 0.051 (*n* = 7) (95% CI: −34.38–745.3)

Data are mean ± SE of the indicated number of mice/group. Statistical significance: * *p* < 0.05, ** *p* < 0.01 or **** *p* < 0.0001 vs. WT female mice, by the one-way ANOVA with the Fisher LSD post-hoc test for multiple comparisons (for a Gaussian distribution), or by the non-parametric Mann-Whitney test (for a non-Gaussian distribution: occasional glycemia, fasting insulin levels, HOMA-IR, HOMA-β, plasma IL-10 and IL-1β levels). HbA_1c_: glycated hemoglobin A_1c_, HOMA-IR: homeostatic model assessment for insulin resistance, HOMA-β: homeostatic model assessment for β-cell function.

**Table 2:** Effect of liraglutide administration on brain cortical hormones’ levels and signaling in female mice with early AD-like pathology.

	WT	3xTg-AD	3xTg-AD + Lira
**Estradiol levels** (pg/mg protein)	5.62 ± 1.19 (*n* = 5) (95% CI: 2.31–8.93)	15.2 ± 2.7 * (*n* = 6) (95% CI: 8.27–22.11)	12.2 ± 3.3 (*n* = 5) (95% CI: 3.1–21.24)
**GLP-1 levels** (pg/mg protein)	5.9 ± 2.5 (*n* = 5) (95% CI: −1.14–12.94)	21.1 ± 6.5 * (*n* = 6) (95% CI: 4.52–37.74)	15.0 ± 2.8 (*n* = 5) (95% CI: 7.29–22.76)
**Active PKA kinase** (ng active PKA/mg protein)	0.01 ± 0.004 (*n* = 6) (95% CI: −0.0005–0.02)	0.001 ± 0.0004 ** (*n* = 6) (95% CI: 0.0001–0.002)	0.009 ± 0.005 (*n* = 5) (95% CI: −0.0048–0.022)

Data are mean ± SE of the indicated number of mice/group. Statistical significance: * *p* < 0.05, ** *p* < 0.01 vs. WT mice, by the one-way ANOVA with the Fisher LSD or Games-Howell post-hoc tests for multiple comparisons (for a Gaussian distribution), or with the non-parametric Mann-Whitney test (for a non-Gaussian distribution: brain estradiol levels and active PKA kinase).

## Supplementary Materials

Supplementary materials can be found at https://www.mdpi.com/1422-0067/21/5/1746/s1.
