# Supplementary material for: Liraglutide Protects Against Brain Amyloid-β1–42 Accumulation in Female Mice with Early Alzheimer’s Disease-Like Pathology by Partially Rescuing Oxidative/Nitrosative Stress and Inflammation
Source: Int J Mol Sci. 2020 Mar 4;21(5):1746. doi: 10.3390/ijms21051746 (PMC7084254; doi:10.3390/ijms21051746)
Supplement: Supplementary file 1 [file ijms-21-01746-s001.pdf]

Supplementary Figure

A.

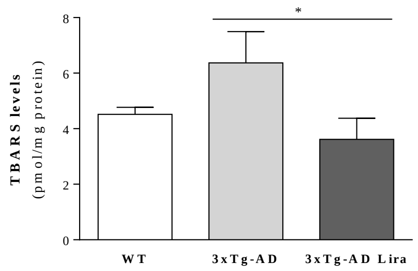

B.

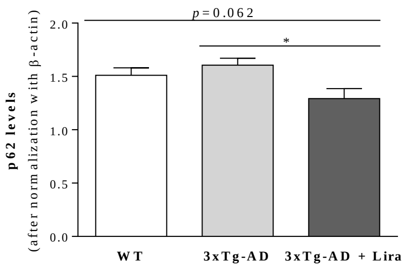

Supplementary Figure 1. Effect of liraglutide on brain cortical lipid oxidation and p62 markers in female mice with early AD-like pathology. Brain cortical TBARS (A) and p62 protein levels (B, after reprobing from membranes labeled to OPA1) were determined. Data are the mean ± SE from 5-6 mice/group. Statistical significance: $^{*}p<0.05$, by the one-way ANOVA with the Fisher LSD post-hoc test for multiple comparisons.
